# Dietary calcium intake is associated with serum high-sensitivity C-reactive protein level in the general Japanese population

**DOI:** 10.3164/jcbn.17-48

**Published:** 2017-11-11

**Authors:** Sakurako Katsuura-Kamano, Hirokazu Uemura, Miwa Yamaguchi, Mariko Nakamoto, Tirani Bahari, Keisuke Miki, Masashi Ishizu, Fusakazu Sawachika, Kokichi Arisawa

**Affiliations:** 1 Department of Preventive Medicine, Institute of Biomedical Sciences, Tokushima University Graduate School, 3-18-15 Kuramoto-cho, Tokushima 770-8503, Japan; 2 Department of Nutritional Science and Metabolism, National Institutes of Biomedical Innovation, Health and Nutrition, 1-23-1 Toyama, Shinjuku-ku, Tokyo 162-8636, Japan; 3 Department of Public Health and Applied Nutrition, Institute of Biomedical Sciences, Tokushima University Graduate School, 3-18-15 Kuramoto-cho, Tokushima 770-8503, Japan

**Keywords:** dietary calcium, C-reactive protein, inflammation, Japanese, cross-sectional studies

## Abstract

The beneficial effects of dietary calcium intake on high-sensitivity C-reactive protein levels, a risk factor of cardiovascular disease, have not been fully elucidated. This study investigated the associations between dietary calcium intake and serum high-sensitivity C-reactive protein levels in the general Japanese population. We analyzed the data of 2,019 subjects (1,194 men and 825 women) aged 35 to 69 years in a cross-sectional study of the Japan Multi-Institutional Collaborative Cohort Study. Nutrients intake including calcium were estimated using a validated food-frequency questionnaire. Analysis using a general linear model revealed that dietary calcium intake was inversely associated with serum high-sensitivity C-reactive protein levels (*p* for trend <0.001) after adjustment for age, sex, research group, leisure-time physical activity, smoking habit, drinking habit, dietary intakes (energy, dietary fiber, saturated fatty acids and vitamin D) and menopausal status. The association was slightly attenuated after additional adjustment for body mass index; however, remained significant (*p* for trend = 0.008). There were no significant interactions between dietary calcium intakes and sex, body mass index, or vitamin D intake for high-sensitivity C-reactive protein levels. This study have demonstrated that dietary calcium intake was inversely associated with serum high-sensitivity C-reactive protein levels in the general population.

## Introduction

Low-grade systemic inflammation is considered a risk factor for the development and progression of inflammation-related disorders, such as cardiovascular disease (CVD)^(1,2)^ and metabolic syndrome.^(3)^ High-sensitivity C-reactive protein (hs-CRP) is a useful marker of inflammation that is able to predict the risk of CVD.^(4)^ Higher CRP levels are related to older age,^(5)^ obesity,^(6)^ smoking,^(7)^ none or high alcohol intake^(8)^ and low physical activity.^(9)^ Nutrient factors, such as dietary fiber^(10)^ and *n*-3 polyunsaturated fatty acids (*n*-3 PUFAs), are inversely associated with hs-CRP levels.^(11)^ In contrast, saturated fatty acids is positively associated with hs-CRP level.^(12)^ Dietary factors including dietary patterns are also associated with hs-CRP levels.^(13,14)^

In previous reports, dietary calcium intake was associated with reduced risk of CVD.^(15–17)^ However, few reports have investigated the association between calcium intake and hs-CRP levels. A randomized-controlled trial (RCT) reported no association between calcium supplementation and hs-CRP levels.^(18)^ Calcium supplementation in vitamin D-insufficient type 2 diabetics also showed no significant change in serum hs-CRP levels compared with placebo.^(19)^ In a cross-sectional study, hs-CRP levels were not significantly different between groups with low and high intake of dietary calcium;^(20)^ however, the results were derived from a relatively small number of subjects (low: *n* = 32 vs high: *n* = 44). Dietary calcium reduces body weight by preventing lipid accumulation in adipocytes,^(21)^ and obesity is associated with inflammatory status.^(22)^ Thus, it is necessary to consider the effect of obesity as an intermediate variable when evaluating the association between calcium intake and hs-CRP levels.

The present study investigated the association between dietary calcium intake and hs-CRP levels in the Japanese population. We also examined the effect of BMI on the association between dietary calcium intake and hs-CRP levels.

## Materials and Methods

### Study subjects

A total of 2,440 participants aged 35 to 69 years, were enrolled in the cross-sectional survey of the Japan Multi-Institutional Collaborative Cohort (J-MICC) Study in Tokushima prefecture. In brief, the J-MICC Study is designed to detect and confirm gene-environment interactions for lifestyle-related diseases.^(23)^ We applied three methods for recruitment of subjects. First, we recruited 570 participants undergoing medical check-ups at the Tokushima Prefectural General Health Check-up Center from January 23, 2008 to November 24, 2011. Second, we mailed companies in Tokushima, inviting them to take part in this research. From November 2009 to June 2012, we recruited 1,174 employees of these companies. They were mostly office workers rather than shift workers. Third, we disseminated approximately 98,700 leaflets explaining the objective and method of the J-MICC Study at each mailbox in Tokushima city (total population = 264,500). After reading the leaflets, 696 subjects voluntarily participated in this study from July 2012 to February 2013. This study was conducted according to the guidelines laid down in the Declaration of Helsinki and all procedures involving human subjects were reviewed and approved by the Ethics Committees of Nagoya University School of Medicine (affiliated with the former principal investigator, Nobuyuki Hamajima), Aichi Cancer Center (affiliated with the current principal investigator, Hideo Tanaka) and Tokushima University Hospital. Written informed consent was obtained from each subject.

Of 2,440 participants, 421 were excluded owing to the following reasons: (i) lack of hs-CRP data (*n* = 29), body mass index (BMI) data (*n* = 12), data regarding physical activity (*n* = 27), and menopausal status (*n* = 1); (ii) hs-CRP levels ≥10 mg/L (*n* = 28); (iii) history of cancer (*n* = 109), ischemic heart diseases (*n* = 51), cerebrovascular disease (*n* = 31), and other inflammatory or chronic diseases (*n* = 23, chronic intestinal diseases and connective tissue diseases); (iv) taking anti-inflammatory drugs (*n* = 77), calcium supplement (*n* = 56), and vitamin D supplements (*n* = 43); (v) implausible estimated total energy intake values (<1,000 kcal/day, *n* = 1 or >4,000 kcal/day, *n* = 17). A total of 2,019 subjects (1,194 men and 825 women) were eligible for the analyses.

### Dietary assessments

A validated food-frequency questionnaire (FFQ), which was developed by Nagoya City University Graduate School of Medical Sciences, asked about the intake frequency of 47 foods and beverages over the past one year.^(24,25)^ The daily intake of total energy, calcium, total dietary fiber, saturated fatty acids, and vitamin D were calculated using an original program^(24)^ based on the Standard Tables of Food Consumption^(26)^ in Japan. The validity of nutrient intake in Aichi prefecture including calcium (energy-adjusted Pearson correlation coefficient was 0.42) was previously reported.^(25)^ In addition, the energy-adjusted Pearson correlation coefficient compared with four season, three-day dietary records in 28 subjects in Tokushima prefecture was 0.43. Dietary calcium, total dietary fiber, saturated fatty acids, vitamin D, and *n*-3 PUFAs were log-transformed because of following right-skewed distributions, and adjusted for energy intake (log-transformed) by using the residual method.^(27)^ They were divided into quartiles before analyses to be similar number of subjects in each category.

### Questionnaires and measurements of hs-CRP levels

Lifestyle factors including smoking and drinking habits, physical activity, current medication, and past history of diseases were investigated by a self-administered questionnaire, and data were checked by trained staff. Smoking and drinking habits were classified into three (never, former, and current smokers) and two (never and former, and current drinkers [≥one time/month]) categories, respectively. Leisure-time physical activity was estimated by multiplying the frequency and average duration of light (walking, hiking, etc., 3.4 metabolic equivalents [METs]), moderate (jogging, swimming, etc., 7.0 METs), and vigorous intensity (marathon running, combative sports, etc., 10.0 METs) exercise, and MET-hours/week of leisure time activity were calculated by summing exercise performed at each level. Subjects were divided into four groups by quartiles of MET-hours/week.

Height (cm) and weight (kg) were obtained, and BMI was calculated as weight (kg)/[height (m)]^2^. Venous blood samples were collected from each subject, and serum hs-CRP levels were measured using a latex agglutination immunoassay (BML Inc., Tokyo, Japan).

### Statistical analysis

For continuous variables of characteristics, a general linear model or Kruskal-Wallis test were applied to assess differences among quartiles of calcium intake, and χ^2^ test was used for categorical variables. Since serum hs-CRP levels followed right-skewed distributions, they were log-transformed before analyses. To analyze associations between dietary calcium intake and serum hs-CRP levels, a general linear model was used adjusting for age (categories: 35–49, 50–59 and 60–69 years), sex, research group (three), leisure-time physical activity (MET-hours/week; quartiles), smoking habit, drinking habit, and dietary intakes (total energy, total dietary fiber, saturated fatty acids and vitamin D; quartiles). Menopausal status (no status [men], premenopausal women and postmenopausal women) was also adjusted instead of sex. Furthermore, we additionally adjusted for BMI (quartiles). Linear trends were assessed using ordinal categorical variables (1 to 4) in each statistical model. Calcium-sex, or -BMI, or -vitamin D interactions on hs-CRP levels were evaluated by including interaction terms of dietary calcium intake (≤median, >median) × sex, or × BMI (≤median, >median), or × vitamin D (≤median, >median) in the model, using partial F-tests. All analyses were performed using the SAS software package (ver. 9.3 for Windows; SAS Institute, Cary, NC). *P* values <0.05 were considered statistically significant.

## Results

Table 1 shows the characteristics of the study subjects according to calcium intake. Compared with subjects in the lowest quartile of dietary calcium, those in the upper quartile were older, had lower BMI, were less likely to be men, current smoker and current drinker, and were physically more active. For nutrition intake, total energy intake was not significantly different among quartiles; however, as calcium intake increased, carbohydrate intake decreased, while protein, fat, saturated fatty acids, total dietary fiber, and vitamin D intakes increased. Higher intakes of calcium were associated with lower serum hs-CRP levels.

Table 2 presents the association between dietary calcium and hs-CRP levels taking into account potential confounding factors. In a general linear model adjusted for age and sex, dietary calcium intake was significantly inversely associated with serum hs-CRP levels (Model 1: *p* for trend <0.001). The significance remained unchanged after adjusting for additional potential confounding factors such as total energy intake, research group, physical activity, smoking habit, drinking habit, menopausal status and intakes of total dietary fiber, saturated fatty acids and vitamin D (Model 2: *p* for trend <0.001). When saturated fatty acid intake and *n*-3 PUFAs intake were included in the same model, multicollinearity was present. Therefore, we adjusted *n*-3 PUFAs intake instead of saturated fatty acid intake in model 2, so that the association was slightly attenuated but remained significant (*p* for trend = 0.02; Table 2 did not show). Because it has been reported that calcium intake is associated with reduced obesity and obesity is positively associated with hs-CRP levels, we further adjusted for BMI, with similar results (Model 3: *p* for trend = 0.008).

Finally, interactions between dietary calcium intake and sex, BMI, or dietary vitamin D on hs-CRP levels were investigated. We did not find significant interaction of calcium × sex (*p* = 0.220), calcium × BMI (*p* = 0.784), or calcium × vitamin D (*p* = 0.150) with hs-CRP levels (Table 3).

## Discussion

To the best of our knowledge, no other reports have observed this inverse association between dietary calcium and hs-CRP levels in the general population. There were only two RCTs^(18,19)^ and a cross-sectional study^(20)^ that evaluated their association, and none of them reported significant association. However, the cross-sectional study included a relatively small number of subjects (low intake of calcium: *n* = 32 vs high intake of calcium: *n* = 44).^(20)^ In addition, the study included only women; there was a relatively high prevalence of obesity, and the subjects had different basal hs-CRP levels and dietary habits (such as different nutritional intake, which affected calcium absorption) compared to those of our subjects. These factors may be responsible for the differences in study results. In mouse models, calcium has been reported to inhibit gene expression of inflammatory cytokine in adipocytes by inhibiting endogenous calcitriol (1,25-dihydroxyvitamin D_3_).^(28)^ Inhibition of calcitriol could have anti-obesity and anti-inflammatory effects by increasing intracellular calcium levels.^(28,29)^ However, this phenomenon has only been reported in mouse adipocytes, and has not been demonstrated in human studies. In a study with human subjects, the group with high calcium intake had lower prevalence of overweight and obesity, but plasma hs-CRP levels and intracellular calcium levels in erythrocyte were not associated with calcium intake.^(20)^ Additional studies are required to further clarify the biological mechanism.

A meta-analysis of prospective cohort studies showed that the association curve between dietary calcium intake and deaths from CVD was U-shaped, and both inadequate and excessive calcium intake were associated with higher risk.^(16)^ The authors further reported that calcium intakes of about 800 mg/day conferred the lowest risk of cardiovascular mortality. The average levels of dietary calcium intakes measured in the present study were 455 mg (energy-adjusted; 448 mg) in men and 509 mg (energy-adjusted; 518 mg) in women. We excluded subjects who consumed calcium supplements to avoid the possibility of excessive intake. The results of our study suggest that the relatively moderate amount of calcium intake from foods in Japanese population might explain the inverse association between dietary calcium and hs-CRP levels. Moreover, Western populations obtain more than 75% of their dietary calcium from dairy products.^(30)^ Likewise, Japanese populations consume 45.3% of the total calcium intake from dairy products, 17.9% from vegetables, 11.4% from beans, 9.7% from fish and shellfish, and 15.7% from other sources.^(31)^ In other words, Japanese populations obtain approximately half of their calcium from non-dairy products. This also may have contributed to the difference in results of Western studies and those of our Japanese study. In the case of an association between dairy products and inflammation, an inverse association was suggested in a meta-analysis of RCTs with overweight and obese subjects.^(32,33)^ However, functional components other than calcium which are present in dairy products, such as milk-derived proteins (e.g., lactoferrin) and bioactive peptides may also exert anti-inflammatory effects.^(33)^ Therefore, it is difficult to assess the effects of calcium on inflammation based on dairy products alone.

There are several reports on the association between calcium and weight loss.^(20,34)^ In the current study, the significant association between dietary calcium and hs-CRP levels remained significant after adjusting for BMI, suggesting that the association was independent of, or not totally mediated by, obesity. Furthermore, the interaction between dietary calcium and BMI on hs-CRP levels was not significant. Thus, the results suggest that the association between dietary calcium intake and hs-CRP did not differ with high or low BMI. Recent studies reported associations between inflammation and type 2 diabetes.^(35)^ To assess this, we further adjusted for history or medication for type 2 diabetes; however, the results did not change (data not shown). The use of statins (for hyperlipidemia), which could have decreased hs-CRP levels,^(36)^ may have confounded the association, although additional adjustment for hyperlipidemia medication status did not change the results (data not shown). It has been reported that activated vitamin D enhances intestinal absorption of calcium;^(37)^ however, the association between calcium and hs-CRP was not affected by vitamin D intake in this study. The biological mechanisms underlying the association between dietary calcium intake and hs-CRP levels remain unclear; thus, future research might help to interpret these observations.

The present study has several limitations. First, since this study used a cross-sectional design, the causal relationships should be discussed with caution. However, we excluded participants with inflammation-related diseases or high hs-CRP levels (≥10 mg/L). Furthermore, participants did not know their hs-CRP status, because it is not usually measured in medical check-ups. Subjects were mostly within the normal range of hs-CRP levels, which suggests that hs-CRP levels might not affect their dietary habits. Second, lifestyle information including dietary habits was self-reported; thus, non-differential misclassification or random measurement errors might have been inevitable. Moreover, because the number of FFQ items (47) was relatively small, the FFQ may have underestimated the absolute level of nutrient intakes. However, FFQs are designed to rank individuals rather than to assess their absolute level of intakes. We also obtained a relatively high correlation coefficient of calcium intake in the validation study (energy-adjusted Pearson r = 0.42).^(25)^ Therefore, the study subjects could be exactly ranked according to calcium intake with reasonable validity. Third, we did not measure other inflammatory biomarkers. Fourth, we did not measure circulating 25-hydroxyvitamin D concentrations, which reflect the actual systemic vitamin D status. Moreover, magnesium intake is also recognized to affect calcium absorption^(38)^ and has been shown to be inversely associated with systemic inflammation.^(39)^ However, our FFQ can only estimate a limited number of nutrients, which does not include magnesium intake. Fifth, because of the observational design of this study, residual confounding remains possible. Finally, further studies in different ethnic groups are necessary because this study included only Japanese populations.

In conclusion, the present study showed a significant inverse relationship between dietary calcium intake and serum hs-CRP concentrations in the general population, an association that was not confounded or totally mediated by BMI. Further studies are needed to confirm the relationship between dietary calcium and inflammation.

## Figures and Tables

**Table 1:** Baseline characteristics of the subjects according to dietary calcium intake^†^

	Calcium intake (mg/day)				*p* value
	Q1 (≤371.5)	Q2 (>371.5 and ≤451.6)	Q3 (>451.6 and ≤548.1)	Q4 (>548.1)	
*n*	505	505	505	504	
Men (%)^‡^	375 (74.3)	335 (66.3)	269 (53.3)	215 (42.7)	<0.001
Age (years)^§^	48.3 ± 9.0	49.1 ± 9.4	49.8 ± 9.2	52.9 ± 9.5	<0.001
BMI (kg/m^2^)^¶^	23.4 (21.6, 25.9)	23.2 (21.2, 25.7)	22.7 (20.9, 25.1)	22.3 (20.7, 24.4)	<0.001
Smoking habit^‡^					
Current	168 (33.3)	113 (22.4)	80 (15.8)	55 (10.9)	<0.001
Past	139 (27.5)	148 (29.3)	124 (24.6)	113 (22.4)	
Never	198 (39.2)	244 (48.3)	301 (59.6)	336 (66.7)	
Drinking habit^‡^					
Current	319 (63.2)	322 (63.8)	288 (57.0)	265 (52.6)	<0.001
Past or Never	186 (36.8)	183 (36.2)	217 (43.0)	239 (47.4)	
Physical activity level (MET-hs/week)^¶^	3.0 (0, 11.6)	3.9 (0.4, 15.4)	4.3 (1.3, 15.3)	7.5 (1.3, 20.4)	<0.001
Nutrients					
Total energy intake (kcal/day)^§^	1,671 (1,467, 1,955)	1,673 (1,487, 1,889)	1,673 (1,491, 1,884)	1,681 (1,499, 1,884)	0.999
Carbohydrate (energy %)^§^	68.5 ± 6.6	65.8 ± 6.8	63.5 ± 7.0	59.7 ± 7.4	<0.001
Protein (energy %)^§^	11.0 ± 1.4	11.9 ± 1.5	12.4 ± 1.6	13.3 ± 1.7	<0.001
Fat (energy %)^§^	20.5 ± 5.6	22.3 ± 5.8	24.1 ± 6.0	27.0 ± 6.2	<0.001
Saturated fatty acids (g/day)^†^^,^^§^	9.0 ± 1.3	9.8 ± 1.4	11.3 ± 2.0	13.4 ± 2.8	<0.001
Dietary fiber (g/day)^†^^,^^§^	8.0 ± 1.9	9.2 ± 2.0	9.8 ± 2.5	11.4 ± 3.1	<0.001
Vitamin D (µg/day)^†^^,^^§^	5.1 ± 1.8	6.1 ± 2.3	6.3 ± 2.5	7.1 ± 3.1	<0.001
hs-CRP (mg/L)^¶^	0.36 (0.18, 0.77)	0.33 (0.15, 0.73)	0.31 (0.16, 0.61)	0.25 (0.13, 0.55)	<0.001

Q, quartiles; BMI, body mass index; MET, metabolic equivalent; hs-CRP, high-sensitivity C-reactive protein. ^†^Adjusted for total energy intake after log-transformation using the residual method. Data were presented as number (%)^‡^, mean ± SD^§^, or median (25%, 75%)^¶^. Differences were analyzed by chi-square test^‡^, general linear model^§^, or Kruskal-Wallis test^¶^.

**Table 2:** Multivariate-adjusted mean values of serum hs-CRP by quartiles (Q1–Q4) of calcium intake

	Q1		Q2		Q3		Q4	*p* for trend
	(≤371.5 mg/day)		(>371.5 and ≤451.6 mg/day)		(>451.6 and ≤548.1 mg/day)		(>548.1 mg/day)	
	Adjusted Means (95% CI)		Adjusted Means (95% CI)		Adjusted Means (95% CI)		Adjusted Means (95% CI)	
Model 1^†^	0.37 (0.33–0.41)		0.35 (0.31–0.38)		0.33 (0.30–0.36)		0.29 (0.27–0.32)	<0.001
Model 2^‡^	0.37 (0.33–0.42)		0.34 (0.30–0.38)		0.32 (0.28–0.35)		0.27 (0.24–0.31)	<0.001
Model 3^§^	0.37 (0.33–0.41)		0.35 (0.31–0.39)		0.33 (0.30–0.36)		0.30 (0.26–0.33)	0.008

hs-CRP, high-sensitivity C-reactive protein; Q, quartiles; CI, confidence interval; BMI, body mass index. ^†^Adjusted for sex and age. ^‡^Adjusted for age, total energy intake, research group, physical activity, smoking habit, drinking habit, total fiber intake, saturated fatty acids intake, vitamin D intake and menopausal status. ^§^Adjusted for variables in model 2 plus BMI.

**Table 3:** Combined effects of calcium intake and sex, BMI or vitamin D intake on serum hs-CRP levels (mg/L)

	Calcium ≤451.6 mg/day (median)		Calcium >451.6 mg/day	*p *for interaction
	Adjusted Means (95% CI)		Adjusted Means (95% CI)	
Men	0.42 (0.39–0.47)		0.36 (0.33–0.40)	0.220^†^
Women	0.31 (0.28–0.36)		0.30 (0.27–0.34)	
BMI ≤22.9 kg/m^2^ (median)	0.26 (0.23–0.29)		0.23 (0.21–0.26)	0.784^‡^
BMI >22.9	0.48 (0.43–0.53)		0.42 (0.38–0.47)	
Vitamin D ≤5.12 µg/day (median)	0.35 (0.32–0.39)		0.30 (0.27–0.33)	0.150^§^
Vitamin D >5.12	0.35 (0.31–0.39)		0.33 (0.30–0.37)	

BMI, body mass index; hs-CRP, high-sensitivity C-reactive protein; CI, confidence interval. ^†^Adjusted for age, total energy intake, research groups, physical activity, smoking habit, drinking habit, total fiber intake, saturated fatty acids intake, vitamin D intake and BMI. ^‡^Adjusted for age, total energy intake, research groups, physical activity, smoking habit, drinking habit, total fiber intake, saturated fatty acids intake, vitamin D intake and menopausal status. ^§^Adjusted for age, total energy intake, research groups, physical activity, smoking habit, drinking habit, total fiber intake, saturated fatty acids intake, menopausal status and BMI.

## Abbreviations

CI: confidence interval
CVD: cardiovascular disease
BMI: body mass index
FFQ: food-frequency questionnaire
hs-CRP: high-sensitivity C-reactive protein
J-MICC Study: Japan Multi-Institutional Collaborative Cohort Study
MET: metabolic equivalent
PUFA: polyunsaturated fatty acid
Q: quartiles
RCT: randomized-controlled trial
